# Automatic Removal of Cardiac Interference (ARCI): A New Approach for EEG Data

**DOI:** 10.3389/fnins.2019.00441

**Published:** 2019-05-08

**Authors:** Gabriella Tamburro, David B. Stone, Silvia Comani

**Affiliations:** ^1^BIND – Behavioral Imaging and Neural Dynamics Center, University “G. d’Annunzio” of Chieti-Pescara, Chieti, Italy; ^2^Department of Neuroscience, Imaging and Clinical Sciences, University “G. d’Annunzio” of Chieti-Pescara, Chieti, Italy

**Keywords:** automatic artifact removal, cardiac artifact, cardiovascular artifact, EEG, ICA

## Abstract

EEG recordings are generally affected by interference from physiological and non-physiological sources which may obscure underlying brain activity and hinder effective EEG analysis. In particular, cardiac interference can be caused by the electrical activity of the heart and/or cardiovascular activity related to blood flow. Successful EEG application in sports science settings requires a method for artifact removal that is automatic and flexible enough to be applied in a variety of acquisition conditions without requiring simultaneous ECG recordings that could restrict movement. We developed an automatic method for classifying and removing both electrical cardiac and cardiovascular artifacts (ARCI) that does not require additional ECG recording. Our method employs independent component analysis (ICA) to isolate data independent components (ICs) and identifies the artifactual ICs by evaluating specific IC features in the time and frequency domains. We applied ARCI to EEG datasets with cued artifacts and acquired during an eyes-closed condition. Data were recorded using a standard EEG wet cap with either 128 or 64 electrodes and using a novel dry electrode cap with either 97 or 64 dry electrodes. All data were decomposed into different numbers of components to evaluate the effect of ICA decomposition level on effective cardiac artifact detection. ARCI performance was evaluated by comparing automatic ICs classifications with classifications performed by experienced investigators. Automatic and investigator classifications were highly consistent resulting in an overall accuracy greater than 99% in all datasets and decomposition levels, and an average sensitivity greater than 90%. Best results were attained when data were decomposed into a fewer number of components where the method achieved perfect sensitivity (100%). Performance was also evaluated by comparing automatic component classification with externally recorded ECG. Results showed that ICs automatically classified as artifactual were significantly correlated with ECG activity whereas the other ICs were not. We also assessed that the interference affecting EEG signals was reduced by more than 82% after automatic artifact removal. Overall, ARCI represents a significant step in the detection and removal of cardiac-related EEG artifacts and can be applied in a variety of acquisition settings making it ideal for sports science applications.

## Introduction

Electroencephalography (EEG) and magnetoencephalography (MEG) are widely used non-invasive methods for measuring brain activity, however, the potential recorded at the scalp is frequently contaminated by additional external and physiological noise. As this interference remains a pervasive problem in EEG and MEG research and clinical applications, there is a continuing need to develop effective techniques to minimize or eliminate artifacts (Urigüen and Garcia-Zapirain, 2015; Mannan et al., 2018). One of the sources of artifactual contamination frequently encountered in EEG/MEG data is due to cardiac activity (Haumann et al., 2016).

To date, there have been multiple methods designed to identify and remove artifacts arising from cardiac sources. Cardiac-related artifacts possess highly stereotypic frequency and waveform characteristics, and methods developed to remove cardiac artifacts exploit these characteristics to identify cardiac interference and remove it from EEG and MEG data. This is accomplished either by monitoring cardiac activity directly during data acquisition (i.e., through electrocardiography, ECG) and/or by exploring the temporal and spectral features of EEG data to detect potential artifactual elements (Al-Qazzaz et al., 2017; Radüntz et al., 2017; Waser et al., 2018). A variety of techniques has been employed to detect cardiac interference, including blind source separation methods (e.g., independent component analysis – ICA) (Barbati et al., 2004), template matching algorithms (Kumar and Reddy, 2016), and spectral analyses (Castellanos and Makarov, 2006; Mammone and Morabito, 2014). However, many contemporary methods utilize a combination of techniques to improve cardiac artifact detection (Wang et al., 2013; Dora and Biswal, 2016; Kumar and Reddy, 2016; Patel et al., 2017; Dora and Biswal, 2019).

Several methods have been successful in removing cardiac interference from EEG and MEG data (Viola et al., 2009; Sun et al., 2016; Mannan et al., 2018), however, there are still several outstanding issues which present challenges to the field. Although some methods work well in specific contexts (Urrestarazu et al., 2004; Abtahi et al., 2014; Hamaneh et al., 2014; Navarro et al., 2015), wide-spread application across multiple acquisition platforms and conditions remains to be evaluated. Additionally, many methods attempt to model cardiac interference based on ECG data acquired concurrently with EEG/MEG data (Zhou and Gotman, 2004; Correa et al., 2007; Devuyst et al., 2008; Dora and Biswal, 2016; Patel et al., 2017), however, while the use of concurrently recorded ECG favored the success of the denoising approach, the need for additional techniques to estimate cardiac interference in EEG/MEG data is a limitation to the general application of these denoising methods. For instance, successful application of methods for the removal of cardiac artifacts and other physiological and non-physiological artifacts from EEG datasets recorded in sports science settings requires that the denoising method is automatic and flexible enough to be applied in a variety of acquisition conditions, that it does not require simultaneous ECG recordings that could restrict movement, and that it is independent from the subjective evaluation of artifactual signal components by expert operators (Reis et al., 2014; Spring et al., 2018).

Another outstanding issue is the presence of cardiovascular artifacts related to blood flow. Current methods generally focus solely on the detection of cardiac interference generated by the electrical activity of the heart, while failing to address additional pulse-related interference which occurs due to the EEG electrode placement near cerebral vasculature. Although pulse-related interference shares the same frequency characteristics as interference due to electrical cardiac activity, it possesses unique temporal and waveform characteristics which may not be captured using current techniques. To our knowledge, there has been only one attempt to eliminate pulse interference from EEG recordings (Waser et al., 2017), however, Waser and colleagues employed concurrently recorded ECG to identify cardio-vascular contamination, and in particular pulse artifacts were grouped together with slow-wave interferences.

We recently developed a method of automatic physiological artifact removal, the Fingerprint Method, which attempted to address some of these issues (Tamburro et al., 2018). The Fingerprint Method employs data decomposition, a set of defined data features optimized to identify multiple artifact types, and a machine learning technique to automatically detect and remove major physiological artifacts. Although the Fingerprint Method proved reliable in the detection and removal of eyeblink, eye-movement, and myogenic artifacts across multiple acquisition platforms and settings, the detection and removal of cardiac-related artifacts met with limited success.

The aim of the present work was to improve upon the Fingerprint Method and develop a stand-alone cardiac-related artifact removal technique capable of detecting cardiac interference across a variety of contexts and settings without requiring simultaneous ECG recording (ARCI – automatic removal of cardiac interference). Our new method, ARCI, defines additional features to better detect and classify cardiac-related artifacts, including potential pulse-related interferences. As with the Fingerprint Method, ARCI focuses on automatic artifact detection and is designed for use in multiple data acquisition settings.

We tested the performance of ARCI using real EEG data acquired in different experimental conditions. Results show that (1) the addition of new features allowed the method to more effectively classify cardiac-related artifacts, (2) the algorithm performed well in detecting and removing both electrical cardiac and pulse-related sources of interference, (3) the proposed method can be utilized under a variety of data acquisition conditions.

## Materials and Methods

### EEG Data

ARCI was tested in two different types of EEG datasets: (1) EEG datasets including externally cued artifacts (cued EEG datasets) that were used previously for validating our Fingerprint Method (Tamburro et al., 2018); (2) EEG datasets recorded during an eyes-closed condition (eyes-closed EEG datasets) at the beginning of a cycling endurance task (Stone et al., 2018).

These EEG datasets were chosen to (1) compare the effectiveness of our new method in removing cardiac-related artifacts with the results obtained using our Fingerprint Method (cued EEG datasets) and (2) to validate ARCI with EEG data collected during simultaneous ECG recording (eyes-closed EEG datasets).

The Ethics Committee of the University “G. d’Annunzio” of Chieti-Pescara (Italy) approved both studies (Ethical Application Ref. n. 10-21/05/2015), and all participants provided written informed consent.

#### Cued EEG Datasets

Cued EEG datasets were recorded from 12 volunteers (male only; age: 28.7 ± 2 years) using an EEG system with a commercial unipolar biosignal amplifier (RefaExt, Advanced Neuro Technologies B.V., Enschede, Netherlands) and a common average reference. EEG signals were acquired at a sampling frequency of 1024 Hz using either a conventional wet electrode cap or a novel dry electrode cap (Fiedler et al., 2015). Both cap types had an equidistant electrodes layout. The commercial wet cap (Waveguard, Advanced Neuro Technologies B.V., Enschede, Netherlands) included 128 Ag/AgCl electrodes whereas the novel dry electrode cap included 97 dry multipin polyurethane electrodes with an Ag/AgCl coating (Fiedler et al., 2015). For both cap types, conventional ring-shaped Ag/AgCl electrodes were applied over the right mastoid with electrolyte gel and served as amplifier ground.

During EEG data acquisition, participants were instructed via a visual cue (computer generated red fixation cross) to generate an eyeblink or a left or right horizontal eye movement with an 8 s inter-trial interval. During acquisition, participants were seated comfortably with their heads fixed in a chin rest to restrict potential head movements. Eyeblink recordings had an average duration of 123 ± 2 s, whereas eye movement recordings had an average duration of 266 ± 3 s. Cued EEG datasets did not include externally recorded ECG signals. Further details of cued EEG datasets acquisitions are provided elsewhere (Tamburro et al., 2018).

Out of a total of 43 cued EEG datasets, 24 datasets were selected because they contained clearly identifiable cardiac artifacts as determined by experienced investigators. Six cued eyeblink and five cued eye movement datasets from the group of dry EEG datasets and six cued eyeblink and seven eye movement datasets from the group of wet EEG datasets were used in the present study.

#### Eyes-Closed EEG Datasets

Eyes-closed EEG datasets were recorded from 16 volunteers (male only; 24.8 ± 3.4 years), who rested with eyes-closed for approximately 2 min to establish a baseline condition prior to an endurance cycling task. Eyes-closed EEG signals were acquired while volunteers remained on the cycle-ergometer without pedaling. During the endurance task, participants cycled on the cycle-ergometer and had to maintain a cycling rate around 80 revolutions-per-minute (RPM) for an average duration of approximately 20 min. The power level was initially set at 50 W and then incrementally increased every 120 s by 25 W during the entire experiment, which ended when the participants reported maximal perceived exertion. Please note that the eyes-closed EEG datasets used in the present study were those acquired prior to the initiation of the endurance task. Further details of the endurance cycling task are provided elsewhere (Stone et al., 2018).

EEG recordings were performed using a unipolar biosignal amplifier at a sampling frequency of 1024 Hz using either a conventional wet electrode cap (Waveguard, Advanced Neuro Technologies B.V., Enschede, Netherlands) or a novel dry electrode cap (eego^TM^sports, Advanced Neuro Technologies B.V., Enschede, Netherlands; Fiedler et al., 2015). Both caps included 64 electrodes arranged in either an adapted 10-10 montage (wet electrode cap) or in a quasi-equidistant montage with an average distance of 30 ± 4 mm between electrode pairs (dry electrode cap). See Supplementary Figure 1 for a layout of the electrode montages from both cap types. Two standard Ag/AgCl electrodes were applied over the left and right mastoid which served as ground electrode and reference electrode, respectively. Wet cap and dry cap EEG datasets were acquired from each subject in separate sessions.

Simultaneous ECG signals were recorded at the same sampling frequency (1024 Hz) during all eyes-closed EEG data acquisitions through a bipolar electrode whose leads were positioned over the left and right fifth intercostal space of the torso.

### Method for Cardiac Interference Identification and Removal

**FIGURE 1:**
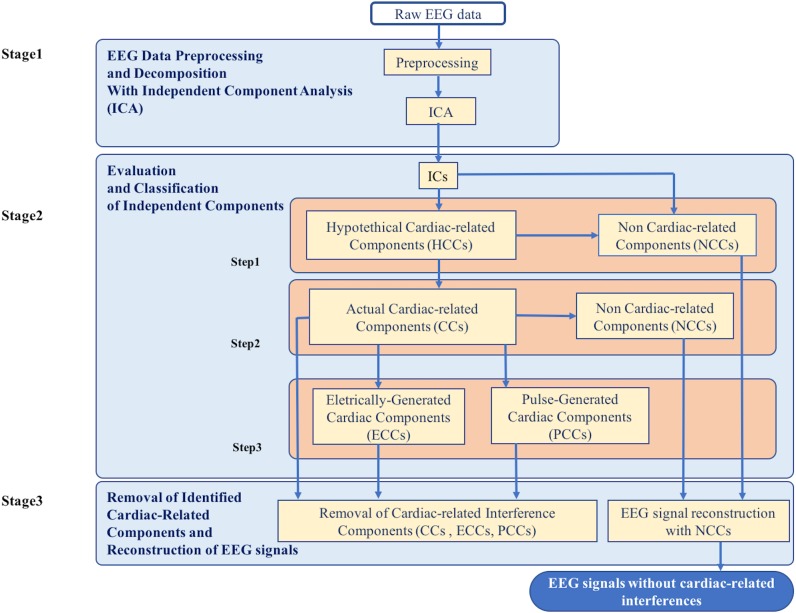
Flowchart of the complete data processing for the automatic classification of cardiac related interference components. The complete data processing pipeline includes three sequential stages of data processing and their respective modular processing steps, input and output.

The new method for the automatic identification and removal of cardiac-related artifacts includes three stages (Figure 1). In stage one, data are preprocessed and decomposed using independent component analysis (ICA). In stage two, the independent components (ICs) separated from each dataset are evaluated for the presence of cardiac-related interference and automatically classified. First, ICs are evaluated and classified as either hypothetical cardiac-related components (HCCs) or as non-cardiac-related components (NCCs). Next, the HCCs are further evaluated and classified as either NCCs or actual cardiac-related components (CCs). Then, if more than one actual cardiac-related component is identified in a given dataset, the set of actual cardiac-related components are further evaluated and classified as either electrically-generated cardiac components (ECCs) or pulse-generated cardiac components (PCCs). In stage three, the ICs classified as containing cardiac-related interference (i.e., the actual cardiac-related components, electrically-generated cardiac components, and pulse-generated cardiac components) are removed, and the EEG signals are reconstructed from the remaining NCCs.

#### Stage 1: EEG Data Preprocessing and Decomposition With Independent Component Analysis (ICA)

All EEG recordings were high-pass filtered at 0.3 Hz and low-pass filtered at 100 Hz, then notch filtered at 50 Hz to remove power line interference. All data were filtered using the FIR filter from the EEGLAB plugin (Widmann et al., 2015).

EEG data segments that contained excessive background noise in more than 50% of the EEG channels, as determined by visual inspection, were removed from the filtered time courses. Typically, these segments consisted of sharp amplitude deflections, probably due to hardware artifacts or a sudden movement of the volunteer, that appeared only in some datasets. The average amount of time removed from any given recording session was 6.81 ± 5.69 s. Then, each EEG channel was inspected, and those channels that exhibited isoelectric saturation or were contaminated by excessive noise during more than the 50% of the time course were removed.

ICA was then applied to effectively parse cardiac-related artifacts from the underlying brain activity. ICA is a statistical blind source separation method that assumes EEG data are a linear mixture of independent non-Gaussian sources except one Gaussian component, which is typically white background noise (Hyvärinen and Oja, 2000; Vigário et al., 2000; Hyvärinen et al., 2001). Of the numerous ICA algorithms available, we chose to use Infomax (available in the EEGLAB toolbox, Bell and Sejnowski, 1995; Delorme and Makeig, 2004) because it has shown good performance in separating artifactual activity from activity generated by brain sources (Karhunen et al., 1997; Lee et al., 1999; Jung et al., 2000; Vorobyov and Cichocki, 2002; Delorme et al., 2007a,b, 2012; Winkler et al., 2011). In particular, we employed the Extended version of Infomax as it is more suitable to separate sources which may possess super-Gaussian and sub-Gaussian distributions (Lee et al., 1999). Prior to ICA decomposition, we performed Principal Component Analysis (PCA) to reduce data dimensionality (Delorme and Makeig, 2004).

With ICA, we decomposed cued and eyes-closed EEG datasets into sets of ICs. To demonstrate the generalizability of ARCI and compare the results to those obtained with the Fingerprint Method, we decomposed each EEG dataset into separate sets of 20 ICs, 50 ICs, and 80 ICs, which mimic different experimental conditions (Tamburro et al., 2018). Given that one ICA requirement is that the number of separated ICs is equal to or less than the number of available channels, pre-processed cued EEG datasets (which had either 97 or 128 channels) were decomposed into 20, 50, and 80 ICs, whereas pre-processed eyes-closed EEG datasets (which had 64 channels) were decomposed into 20 and 50 ICs only. All data pre-processing and ICA decompositions were performed using EEGLAB (release 14.1.1b) operating in the Matlab environment (release MatlabR2016; Mathworks, Natick, MA United States).

#### Stage 2: Evaluation and Classification of Independent Components

The ICs of each dataset were automatically evaluated and each IC was classified based on a set of evaluation criteria. Stage 2 proceeded in several steps, as described in the following sub-sections. At each step, the set of ICs was subdivided into separate classes where each class was further evaluated and classified to produce a final set of classified ICs. The procedure of Stage 2 is outlined in Figure 1.

##### Step 1: classification of hypothetical cardiac-related components and non-cardiac-related components

Interference due to cardiac activity (either electrical or pulse related) generates a periodic waveform at a frequency that corresponds to the cardiac frequency (expressed in beats per minute, BPM). To determine if a given IC possessed cardiac interference, ARCI evaluated its frequency characteristics. First, a cardiac frequency range which spans the range of potential cardiac frequencies was defined. Typical at rest cardiac frequencies vary from 60 to 100 BPM (Opthof, 2000) for healthy individuals and from 40 to 50 BPM for athletes (Azevedo et al., 2014). Given that all datasets were acquired during rest or typical experimental task conditions in the absence of physical activity, the cardiac frequency range was defined as 0.6–1.7 Hz for all volunteers, which corresponds to the interval 36–102 BPM. This frequency range can easily be adjusted to account for conditions where increased heart rates are anticipated, as during sports performance and endurance tasks. Next, the power spectral density (PSD) of the IC was calculated, and the maximum power peak between 0.4 and 99.0 Hz was identified. We excluded peaks in the 0.3–0.4 Hz and 99.0–100 Hz ranges to avoid possible edge effects caused by data filtering [see section Stage 1: EEG Data Preprocessing and Decomposition With Independent Component Analysis (ICA)]. If the maximum power peak for the analyzed IC occurred within the defined cardiac frequency range, this peak was identified as the hypothetical cardiac frequency, and the IC was classified as a HCC and selected for further classification. Otherwise, the IC was classified as a NCC and removed from further classification. If all ICs in an EEG dataset were classified as NCCs, then the analysis ended and the outcome for that dataset was “NO CARDIAC COMPONENTS FOUND.”

##### Step 2: classification of hypothetical cardiac-related components as actual cardiac-related components or non-cardiac-related components

All ICs classified as HCCs in the previous step were further evaluated based on their time courses. Cardiac interferences typically generate a periodic waveform which includes a high amplitude segment (an HA-peak) followed by a low amplitude segment (an LA-peak) of opposite polarity. This waveform corresponds to ventricular depolarization (HA-peak) followed by the initial phases of ventricular repolarization (LA-peak). The time courses of the HCCs from each dataset were evaluated to identify HA-peak and LA-peak segments. First, the hypothetical cardiac frequency of each HCC was used to calculate the inter-beat interval (IBI), corresponding to the period of the hypothetical cardiac frequency (Equation 1):

(1) $$IBI=\frac{1}{\text{Hypothetical Cardiac Frequency}}$$

Next, all positive peaks which occurred at intervals separated by at least 75% of the IBI in the time course of the HCC were selected. We chose to consider the IBIs with a duration greater than the 75% of the defined IBI in order to account for heart rate variability, which can lead to IBIs that are shorter or longer than the calculated IBI. Given that this first selection might include also peaks that are not related to cardiac activity, we proceed to use a second criterion to select only those positive peaks that (1) are separated by an inter-peak interval that might be an IBI and (2) have an amplitude that might correspond to one of the two main waves of the cardiac interference, i.e., the HA-peak or LA-peak. To this purpose, we calculated the mean amplitude of all selected positive peaks, and identified a sub-group of peaks that satisfy both conditions: (1) the peaks occurred at intervals separated by at least 75% of the IBI; (2) the peaks concurrently had an amplitude greater than 50% of the mean peak amplitude. The same procedure was repeated with all negative peaks, so that we finally had two series of peaks: one of positive peaks and one of negative peaks, both related to cardiac activity.

Given that the HA-peak/LA-peak waveform can appear in reversed polarity in the HCC time course, to correctly identify the HA-peak and LA-peak segments, we calculated the mean amplitudes of both positive and negative peak series. Then, the series of peaks with the greatest absolute mean amplitude was identified as the series of HA-peak segments of the cardiac interference waveform, whereas the series of peaks with the lower absolute mean amplitude was identified as the series of LA-peak segments.

Once the two series of HA-peak and LA-peak segments were identified in the HCC time course, two measures of cardiac interference were calculated. The first measure was the Cardiac Identification Feature (CIF) which was first introduced in the Fingerprint Method (Tamburro et al., 2018). The CIF was calculated as the ratio of the actual number of selected HA-peak segments (*N_fcp_*) to the number of HA-peak segments expected based on both the HCC hypothetical cardiac frequency and the HCC time course duration (*N_ecb_*; Equation 2).

(2) $$CIF=\frac{N_{fcp}}{N_{ecb}}$$

Next, we calculated a second measure of cardiac interference in the HCC time courses, the Cardiac Correlation Index (CorrCI), which is based on the fact that, while some variability can exist in the timing of the cardiac-related interference waveform, the HA-peak/LA-peak waveform should remain constant in a given IC. The CorrCI compares the shape of each selected HA-peak with an HA-peak template obtained by averaging a series of 200 ms epochs centered on the identified HA-peak segments in the HCC. A window of 200 ms was selected based on the typical duration of the HA-peak which varies between 60 and 100 ms in healthy individuals (Hnatkova et al., 2016). For each HCC, we calculated the correlation between each HA-peak segment and the HA-peak template. The measure CorrCI was given by the mean correlation value across all HA-peak segments (Equation 3):

(3) $$CorrCI=\frac{\Sigma_{i=1}^{N}{CT}_{i}}{N}$$

where CT*_i_* is the *i*th correlation value between the HA-peak template and the *i*th HA-peak segment, and N is the total number of HA-peak segments in the HCC time course.

Each HCC in an EEG dataset was then classified based on the values of both CIF and CorrCI. If the HCC had a CIF value >0.95 and a CorrCI value >0.55, then it was retained for further evaluation. If only one HCC was classified, no further evaluation was possible, it was considered as a true cardiac-related component and classified as CC. All other components were classified as NCCs. If all HCCs in an EEG dataset were classified as NCCs, then the analysis ended and the outcome for that dataset was “NO CARDIAC COMPONENTS FOUND.”

If more than one HCC was retained, we could further evaluate these components and determine whether they were truly related to cardiac activity. To this end, we performed the following steps to identify the true cardiac frequency (TCF) and select the HCCs that were actual cardiac-related components. Given that the spectrum of a saw-tooth function has peaks only at the fundamental frequency and its harmonics (as the spectrum of a true cardiac-related signal), we calculated the correlation between the PSD spectrum of each retained HCC with the PSD spectrum of a saw-tooth function having the HCC hypothetical cardiac frequency as fundamental frequency. Then, the TCF was identified as the hypothetical cardiac frequency of the HCC that displayed the highest correlation between its PSD and the PSD of the corresponding saw-tooth function among all HCCs.

**FIGURE 2:**
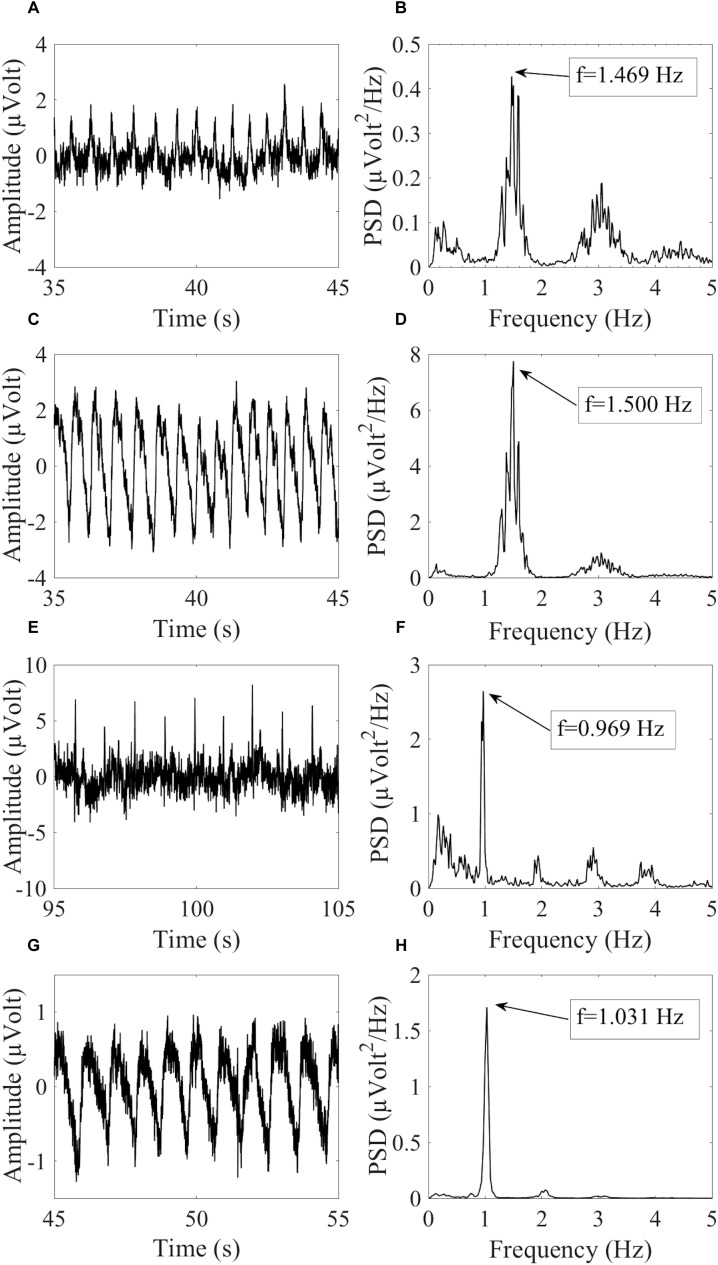
Example of similar cardiac frequencies in ICs including electrically-generated cardiac interference (ECC) and pulse-generated cardiac interference (PCC) from different datasets recorded with the dry cap and the wet cap. In **(A,B)** the time course and PSD spectrum of an ECC separated from a dry cued dataset decomposed in 80 ICs are given. In **(C,D)** the time course and PSD spectrum of a PCC separated from the same dataset are given. Although the two components were separated from the same dataset, they showed slightly different peak frequencies because of the PSD resolution: 1.469 Hz for the ECC and 1.500 Hz for the PCC. In **(E,F)** the time course and PSD spectrum (peak frequency at 0.969 Hz) of an ECC separated from a wet cued dataset decomposed in 50 ICs are given. In **(G,H)** the time course and PSD spectrum (peak frequency at 1.031 Hz) of a PCC separated from another wet cued dataset decomposed in 80 ICs are given.

Once TCF was identified, we could check that all retained HCCs were actual cardiac-related components by verifying that their hypothetical cardiac frequency was close to TCF. The similarity of these frequencies was determined on the basis of the frequency resolution of the PSD spectra according to Equation (4):

(4) $$1-2*(FR)<\frac{f_{{HCC}_{i}}}{TCF}<1+2*\left(FR\right)$$

where f*_HCCi_* is the hypothetical cardiac frequency of the *ith* HCC, TCF is the TCF, and FR is the frequency resolution of the PSD spectra. Figure 2 provides an example of similar cardiac frequencies in a cardiac-electrical IC and a pulse IC.

If an HCC met the condition set out in Equation (4), then it was classified as an actual cardiac-related component (CC), otherwise it was re-classified as an NCC.

##### Step 3: classification of actual cardiac-related components as electrically-generated cardiac components and pulse-generated cardiac components

In the final step of classification, if more than one CC was classified in a dataset, our new method attempted to identify the specific type of cardiac-related interference as either caused by the electrical activity of the heart (electrical cardiac interference – ECC) or by vascular displacement due to blood pressure (pulse-related cardiac interference – PCC). The classification of a CC as ECC or PCC is based on the evaluation of the delay between electrical and pulse cardiac interferences. According to the electrophysiology of the heart and to the blood pressure dynamics, the electrical cardiac interference typically precedes the pulse interference by about 200–300 ms (Benbadis et al., 2012; Kwon et al., 2018; Rajala et al., 2018). Based on this fact, all possible pairs of CCs in a dataset were evaluated. For each pair of CCs, we calculated the time delay between the two CCs through the phase difference of the cross-spectrum between CC_1_ and CC_2_ according to Equation (5):

(5) $$180ms\leq |\phi CC_{1}-\phi CC_{2}|\leq 320ms$$

where ϕCC_1_ is the phase of the cardiac frequency of the 1st CC in the pair and ϕCC_2_ is the phase of the cardiac frequency of the 2nd CC in the pair. If the phase difference of the cross-spectrum was less than 180 ms or greater than 320 ms, then it was not possible to establish whether one CC preceded or followed the other CC and both CCs in the pair retained the CC classification. On the other hand, if ϕCC_2_ lagged ϕCC_1_, the 1st CC in the pair was re-classified as an ECC and the 2nd CC was re-classified as a PCC. If ϕCC_2_ led ϕCC_1_, then the 1st CC was re-classified as a PCC and the 2nd CC was re-classified as an ECC.

#### Stage 3: Removal of Identified Cardiac-Related ICs and Reconstruction of EEG Signals

Stage 2 of our new method concluded with the classification of each IC in each EEG dataset as CC, ECC, PCC, or NCC. In Stage 3, cardiac-related interference components (CCs, ECCs, and PCCs) were removed from each dataset and the EEG signals were reconstructed by re-projecting only the non-cardiac-related interference components (NCCs) back into the sensor space.

### Performance Evaluation

#### Evaluation of CIF and CorrCI Selectivity

The classification of HCCs as CCs and NCCs was based on the calculation of two features, the CIF and the CorrCI. To assess the classification power of each feature, the total number of HCCs classified as CCs by each feature was calculated for all datasets and all decomposition levels.

#### Validation of the Method: Comparison With Experienced Investigator Classifications

To assess the overall performance of ARCI, we compared all IC classifications obtained from the algorithm with the IC classifications made by two independent experienced investigators, who evaluated the spectral and time course properties of all IC components and classified each IC as either a cardiac-related component (CC) or a NCC. Given that the experienced investigators and the automatic method classified the ICs in a different number of classes, to evaluate the overall performance of ARCI in effectively removing all cardiac-related components, all automatically classified cardiac-related interference components (CCs, ECCs, and PCCs) were considered as a unique group of removed cardiac components (RCC). Similarly, the classifications made by the experts, which were either ECC or PCC for the cardiac- or pulse-generated interferences, were merged to form a unique group of RCCs. The automatically classified removed or retained components groups were compared with the same groups classified by the experts.

For each IC, the automatic and investigator classifications were compared and each IC was labeled according to the following criteria. If the automatic and investigator classifications for an IC were the same, then the IC was labeled as a true positive (TP) when both classifications were RCC or as a true negative (TN) when both classifications were NCC. When the IC was classified as a RCC by the experienced investigators but as a NCC by ARCI, the IC was labeled as a false negative (FN). If the IC was classified as a RCC by ARCI but as a NCC by the investigators, the IC was labeled as a false positive (FP).

Three statistical measures were used to evaluate the classification performance of ARCI. First, we calculated the accuracy of the algorithm classifications as the proportion of all true IC classifications in all classified ICs according to Equation (6).

(6) $$Accuracy=\frac{\Sigma \left(TP+TN\right)}{\Sigma \left(TP+TN+FP+FN\right)}\,\;$$

Second, we calculated the false omission rate (FOR) of algorithm classifications as the proportion of ICs falsely labeled as NCC out of all NCC classifications according to equation (7):

(7) $$FOR=\frac{\Sigma FN}{\Sigma \left(TN+FN\right)}$$

Third, we calculated the sensitivity (*p*) of the classification algorithm according to Equation (8) (National Research Council (US) Committee on Vision, 1985; Tamburro et al., 2018):

(8) $$p=\frac{HR-FAR}{1-FAR}$$

where HR is the hit rate, defined in Equation (9), and FAR is the false alarm rate, defined in Equation (10):

(9) $$HR=\frac{\Sigma TP}{\Sigma \left(TP+FN\right)}$$

(10) $$FAR=\frac{\Sigma FP}{\Sigma \left(FP+TN\right)}$$

When the expert and algorithm classifications were in agreement for all ICs, *p* was equal to 1 and FAR was equal to zero.

Accuracy, FOR, and *p* were evaluated separately for the cued EEG datasets and the eyes-closed EEG datasets at each decomposition level.

Although only two classes (RCC and NCC) were applied for validating the performance of the new method, the percentage of each type of cardiac-related components automatically classified by ARCI as ECCs and PCCs was also assessed. By comparing this classification with the one performed by the experienced investigators we could check that ARCI included not only electrical cardiac interference but also pulse related components.

#### Validation of the Method: Comparison With Concurrently Recorded ECG

ECG data were simultaneously recorded via an external bipolar lead during eyes-closed EEG dataset acquisitions, permitting assessment of the effectiveness of our new classification method in these datasets. First, the PSD of the ECG signal for each eyes-closed EEG dataset was calculated from 0.3 to 100 Hz and compared to the PSD of each IC separated from that dataset by measuring the correlation between the PSD of the ECG and the PSD of the IC across the entire frequency spectrum. Then, the correlation values for all ICs automatically classified as RCCs were compared to the correlation values for all ICs automatically classified as NCCs by performing a two-tailed independent samples *t*-test. We expected that, if the automatic IC classification by ARCI was correct, the average correlation value obtained for the RCCs was significantly higher than the average correlation value obtained for the NCCs.

#### Evaluation of EEG Signal Reconstruction Following Artifact Removal

In Stage 3 of our method, the components classified as CC, PCC, or ECC were removed and the EEG time courses were reconstructed by re-projecting only the NCCs back into the sensor space. To evaluate the quality of the EEG signals reconstructed after the automatic removal of cardiac interference, the level of contamination of the signal before and after removal of the cardiac-related components was compared by calculating the signal-to-noise ratio (SNR) across data segments containing electrical and pulse cardiac interferences based on Equation (11) (Tamburro et al., 2018):

(11) $$SNR=10\log_{10}\frac{\left({\text{max signal}}^{2}\right)}{\left({\text{max noise}}^{2}\right)}$$

For the analysis, *signal* was defined as the time segment containing the electrical or pulse cardiac artifact, and *noise* was defined as the 100 ms segment preceding the artifact. A SNR reduction is therefore indicative of an effective artifact removal. SNR was computed separately for the electrically-generated cardiac interference and for the pulse-generated cardiac interference. The electrical signal was defined as a 300 ms segment centered on the HA-peak, whereas the pulse signal was defined as a 400 ms segment centered on the HA-peak. The longer duration of the pulse signal was chosen to capture its slow wave characteristics.

## Results

### Evaluation of CIF and CorrCI Selectivity

**FIGURE 3:**
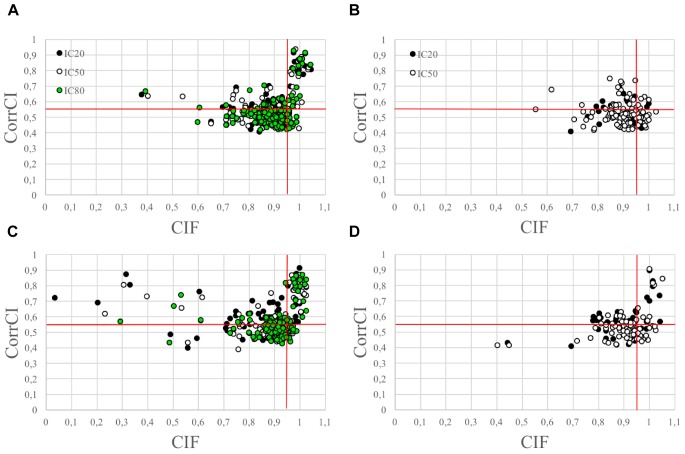
Scatterplots illustrating the HCCs classification performed using CIF and CorrCI. HCCs are classified as CCs if CIF > 0.95 and CorrCI > 0.55. Therefore, only the HCCs included in the upper right quadrant of the scatter plots are classified as CCs. **(A,C)** Refer to cued wet and dry EEG datasets, respectively. **(B,D)** Refer to eyes-closed wet and dry EEG datasets, respectively. The different colors used to identify the HCCs in the scatter plots refer to the decomposition level. The horizontal and vertical red lines indicate the thresholds for CorrCI and CIF, respectively. The total number of HCCs in the scatterplot related to cued wet EEG datasets **(A)** is 345 (i.e., 66 HCCs at 20 ICs decomposition level, 124 HCCs at 50 ICs decomposition level, 155 HCCs at 80 ICs decomposition level). The total number of HCCs in the scatterplot related to cued dry EEG datasets **(C)** is 380 (i.e., 75 HCCs at 20 ICs decomposition level, 120 HCCs at 50 ICs decomposition level, 185 HCCs at 80 ICs decomposition level). The total number of HCCs in the scatterplot related to eyes-closed wet EEG datasets **(B)** is 146 (i.e., 37 HCCs at 20 ICs decomposition level, and 109 HCCs at 50 ICs decomposition level. The total number of HCCs in the scatterplot related to eyes-closed dry EEG datasets **(D)** is 134 (i.e., 47 HCCs at 20 ICs decomposition level, and 87 HCCs at 50 ICs decomposition level).

The use of the CIF feature alone was less effective in correctly classifying the HCCs as CCs than the combined use of CIF and CorrCI. Furthermore, the selectivity of CIF and CorrCI increased at higher decomposition levels, resulting in fewer FPs (Figure 3). In fact, in cued EEG datasets the percentage of HCCs classified as CCs using only the CIF feature was 22.7% for wet and 25.3% for dry datasets at 20 IC decomposition level, whereas it decreased to 16.7 and 24.0%, respectively, when using both features, demonstrating the higher selectivity of the combined use of CIF and CorrCI. At 50 IC decomposition level, the percentage of HCCs classified as CCs using only the CIF feature was 19.4% for wet and 20.0% for dry datasets, compared to 15.3 and 15.0%, respectively, when using both features. At 80 IC decomposition level, the percentage of HCCs classified as CCs using only the CIF feature was 18.1% for wet and 20.0% for dry datasets, compared to 12.9 and 11.4% using both features.

In eyes-closed EEG datasets the percentage of HCCs classified as CCs using only the CIF feature was 29.7% for wet and 29.8% for dry datasets at 20 IC decomposition level, whereas it decreased to 8.1 and 19.1%, respectively, when using both features. At 50 IC decomposition level, the percentage of HCCs classified as CCs using only the CIF feature was 23.8% for wet and 24.1% for dry datasets, compared to 4.6 and 9.2%, respectively, when using both features.

### Validation of the Method: Comparison With Experienced Investigator Classifications

The performance of ARCI in classifying the cardiac related-interferences in the cued and eyes-closed EEG datasets was statistically assessed according to cap type (wet, dry) and decomposition level (20, 50, 80 ICs). The results are summarized in Table 1.

**Table 1:** Outcome of the validation of our new method ARCI by comparing its automatic classifications with those of the experienced investigators.

Dataset type	No. of ICs per dataset	Electrode type	No. of datasets	Total No. of ICs	Total No. of artifactual ICs	True positive	True negative	False positive	False negative	Accuracy	FOR	HR	FAR	Sensitivity *p*
Cued EEG datasets	20	WET	13	260	10	10	249	1	0	0.996	0	1	0.004	1


						(10 CC)		(1 CC)						
		DRY	11	220	19	17	201	0	2	0.991	0.010	0.895	0	0.895
						(5 ECC, 4 PCC, 8 CC)								
	50	WET	13	650	14	14	634	2	0	0.997	0	1	0.003	1
						(1 ECC, 1 PCC, 12CC)		(2CC)						
		DRY	11	550	18	17	531	1	1	0.996	0.002	0.944	0.002	0.944
						(7 ECC, 6 PCC, 5 CC)		(1 ECC)						
	80	WET	13	1040	16	15	1024	0	1	0.999	0.001	0.938	0	0.938
						(15 CC)								
		DRY	11	880	20	19	860	0	1	0.999	0.001	0.950	0	0.950
						(6 ECC, 5 PCC, 8 CC)								
Eyes-closed EEG datasets	20	WET	16	320	2	2	317	1	0	0.997	0	1	0.003	1


						(2 CC)		(1 CC)						
		DRY	16	320	7	7	312	1	0	0.997	0	1	0.003	1
						(1 ECC, 1 PCC, 5 CC)		(1 CC)						
	50	WET	16	800	3	2	794	3	1	0.995	0.001	0.667	0.004	0.665
						(2 CC)		(3 CC)						
		DRY	16	800	7	5	791	2	2	0.995	0.003	0.714	0.003	0.714
						(1 ECC, 1 PCC, 3 CC)		(2 CC)						


*Results given in the first three rows refer to cued EEG datasets at each decomposition level (20, 50, and 80 ICs) and for each cap type (wet and dry). The last two rows include the results in eyes-closed EEG datasets at each decomposition level (20 and 50 ICs) and for each cap type (wet and dry).*

For cued EEG datasets, our method reached an overall accuracy >0.99, FOR ≤ 0.01, HR > 0.89, FAR ≤ 0.004 and sensitivity *p* > 0.89 across cap types and decomposition levels. Both types of recordings (wet and dry) reached the highest accuracy at 80 ICs decomposition level, but small differences can be observed with respect to sensitivity *p*, with highest values (*p* = 1) on wet datasets at lower decomposition levels.

Overall, there were fewer artifactual ICs in the eyes-closed EEG datasets. On these datasets, the method performance across cap types and decomposition levels reached an overall accuracy >0.99, FOR ≤ 0.003, HR > 0.66, FAR ≤ 0.003 and sensitivity *p* > 0.66, with the best performance at the 20 IC decomposition level (accuracy = 0.997, FOR = 0, HR = 1, FAR = 0.003, and *p* = 1).

#### Comparison With Experienced Investigator Classifications: Differentiating ECCs and PCCs

Table 2 displays the total number of cardiac-related ICs classified by type (ECC or PCC) across all cap types and decomposition levels. Note that the experienced investigators classified all cardiac-related components as either ECC or PCC, while ARCI retained an additional classification of artifactual components undifferentiated by type (the CC classification). In cued EEG datasets, the experienced investigators identified a total of 97 cardiac-related components, of which 69 (i.e., 71.1%) were ECCs and 28 (i.e., 28.9%) were PCCs. Since in the automatic classification, where a total of 96 cardiac-related ICs were detected, there were 4 FPs and 5 FNs, the algorithm correctly classified 27.5% of the investigator classified ECCs (i.e., 19 ECCs) and 57.1% of the investigator classified PCCs (i.e., 16 PCCs). Given that, out of the 97 identified cardiac-related components, 16 were correctly classified as PCCs by the algorithm (see Table 1), 19.8% more cardiac-related components were removed due to the PCC classification performed by ARCI. The remaining 61 cardiac components classified as CCs by the algorithm may refer to either electrical or pulse interference.

**Table 2:** Comparison between the ECC and PCC classifications performed by ARCI and by the experienced investigators in the two types of datasets.

Dataset type	Classifier	Total No. of ICs	ECCs	PCCs	CCs	Total No. of RCCs	NCCs
Cued EEG datasets	Experienced investigators	3600	69	28	–	97	3504
			1.92%	0.78%		2.69%	97.33%
	Algorithm	3600	19	16	61	96	3503
			0.53%	0.44%	1.69%	2.67%	97.31%
Eyes-closed EEG datasets	Experienced investigators	2240	6	13	–	19	2221
			0.27%	0.58%		0.85%	99.15%
	Algorithm	2240	2	2	19	23	2217
			0.09%	0.09%	0.85%	1.03%	98.97%


*The percentage values were calculated with respect to the total number of classified ICs. Differences in the percentages may be due to rounding.*

In eyes-closed EEG datasets, the investigators identified a total of 19 cardiac-related components, of which 6 (i.e., 31.6%) were ECCs and 13 (i.e., 68.4%) were PCCs. Given that the automatic method identified a total of 23 artifactual components, and that there were 7 FPs and 3 FNs in the overall classification, ARCI correctly classified 33.3% of the investigator classified ECCs (i.e., 2 ECCS) and 15.4% of the investigator classified PCCs (i.e., 2 PCCS). As with the cued EEG datasets, out of the 19 identified cardiac-related components, 2 were correctly classified as PCCs by ARCI (see Table 1), corresponding to 11.8% more cardiac-related components that were removed due to the PCC classification. The remaining 19 cardiac components classified as CCs by the algorithm may refer to either electrical or pulse interference.

### Validation of the Method: Comparison With Concurrently Recorded ECG

To compare the classification performance of ARCI with externally recorded ECG, for each eyes-closed EEG dataset we computed the correlation between the PSD of the ECG signal and the PSD of each automatically classified RCC or NCC (see section Step 2: classification of hypothetical cardiac-related components as actual cardiac-related components or non-cardiac-related components). Correlations in the two groups (RCCs and NCCS) were compared by examining mean differences using a two-tailed independent samples *t*-test (Table 3). Correlations between the PSDs of the ECGs and the RCCs were significantly greater than the correlations between the PSDs of the ECGs and the NCCs [*t*(22.16) = 7.87, *p* < 0.001].

**Table 3:** Validation of ARCI classification performance in eyes-closed EEG datasets with concurrently recorded ECG.

		Descriptive statistics of PSD correlation values					
			
Type of ICs	Total N. of ICs	Mean	SD	Median	Interquartile	95° percentile	*p*(*t*-test; two tails)
CCs	23	0.67	0.26	0.79	0.31	0.92	
(2 ECCs, 2PCCs, 19 CCs)							<0.001
NCCs	2067	0.24	0.15	0.20	0.17	0.55	


*The descriptive statistics of the correlation values between the PSDs of the ECG signals and the PSDs of the classified ICs (as either CCs or NCCs) refers to both cap types and all decomposition levels (20 and 50 ICs). The result of t-test between the mean values of the two correlation value groups is given in the last column.*

### Evaluation of EEG Signal Reconstruction Following Artifact Removal

**FIGURE 4:**
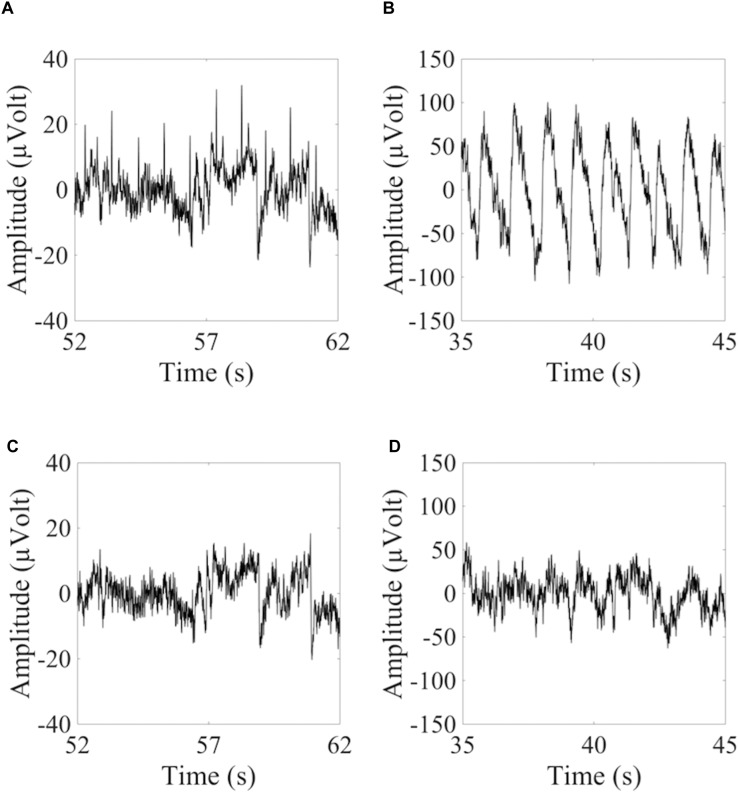
Examples of 10 sec from the time course of EEG signals containing cardiac related artifacts. **(A,C)** Refer to the signal recorded by electrode LE4 of a wet cued EEG dataset before **(A)** and after **(C)** removal of the automatically classified ECCs. **(B,D)** Refer to the signal recorded by electrode 2R of a dry eyes-closed EEG dataset before **(B)** and after **(D)** removal of the automatically classified PCCs.

To show how signal quality changes due to the removal of cardiac artifacts, we selected 10 exemplary time segments from one cued wet EEG dataset where cardiac electrical interference could be observed by visual inspection (Figure 4A). Only the ICs classified as ECCs by ARCI were removed, and the EEG signals were reconstructed from the remaining ICs. SNR was calculated for the three decomposition levels (20 ICs, 50 ICs, and 80 ICs) for each time segment before and after ECCs removal. On average, the relative reduction in SNR following ECCs removal was greater than 87%, indicating an effective reduction of cardiac interference (i.e., the *signal*) in the EEG recordings (Table 4). Figure 4C shows the exemplary EEG trace containing electrical cardiac interference after ECCs removal.

**Table 4:** Average SNR values of exemplary EEG signals before and after removal of the cardiac-related artifacts.

Artifact type	Dataset type	SNR in filtered EEG (dB)	SNR in artifact-free EEG (dB)			Relative SNR reduction (%)		
			
			20 ICs	50 ICs	80 ICs	20 ICs	50 ICs	80 ICs
Electrically-generated	Cued EEG (wet cap)	11.9 ± 2.1	1.6 ± 1.1	1.5 ± 1.2	1.4 ± 1.1	86.9 ± 8.8	87.2 ± 9.5	88.0 ± 9.1
Pulse-generated	eyes-closed EEG (dry cap)	13.3 ± 2.0	1.8 ± 0.9	2.4 ± 1.4	–	82.3 ± 9.7	84.2 ± 11.0	–


*Electrically-generated cardiac interference: the SNR was calculated for the EEG signal recorded by electrode LE4 of a cued wet EEG dataset before and after removal of all automatically classified ECCs for the three decomposition levels (20, 50, and 80 ICs). Pulse-generated cardiac interference: the SNR was calculated for the EEG signal recorded by electrode 2R of a eyes-closed dry EEG dataset before and after removal of all automatically classified PCCs for the two decomposition levels (20 and 50 ICs).*

Similarly for pulse artifacts, 10 time segments from a eyes-closed EEG dataset where pulse interference could be observed by visual inspection (Figure 4B) were selected. Only PCCs automatically classified by ARCI were removed, and the EEG signals were reconstructed from the remaining ICs. SNR was calculated for the two decomposition levels (20 ICs and 50 ICs) before and after PCCs removal. On average, the relative reduction in SNR following PCCs removal was greater than 82%, indicating, as for the cardiac electrical interference, that the pulse artifacts (i.e., the *signal*) were effectively removed from the EEG recordings (Table 4). Figure 4D shows the exemplary EEG trace containing pulse interference after ECCs removal.

## Discussion

Cardiac interference is one of the most difficult artifacts to remove from EEG and MEG data. In particular when applying EEG in sports science, it is crucial that the cardiac artifact removal approach adopted is flexible to permit EEG application in a variety of acquisition conditions, does not require simultaneous ECG recording, and is automatic in order to be independent from the subjective evaluation of expert operators (Reis et al., 2014; Spring et al., 2018). In this study, we developed a new ICA-based method for the automatic removal of cardiac-related interference (ARCI) from EEG recordings without the need for simultaneous ECG. Our new method used features calculated in the time and frequency domains to classify ICs of likely cardiac origin, which include artifacts due to the electrical activity of the heart and to cardiovascular dynamics.

Results indicate that ARCI performed well in classifying cardiac-related artifacts when compared to classifications made by experienced investigators. Indeed, the overall accuracy of the algorithm in artifact classification was greater than 99% in all datasets evaluated (varying from 99.1 to 99.9% as a function of the type of electrodes and decomposition level), while the average sensitivity was greater than 0.90 (varying from 0.895 to 0.950, as a function of the type of electrodes and decomposition level). In our previous work, we developed an algorithm for the automatic classification of multiple types of physiological artifacts, including cardiac-related artifacts (the Fingerprint Method; Tamburro et al., 2018). While the Fingerprint Method performed well in classifying eyeblink, eye movement, and muscle artifacts, performance during cardiac artifact classification was generally poor, resulting in an average accuracy of 97.2% and an average sensitivity of only 0.19. In the present study, we evaluated a subset of the same datasets that were used for the Fingerprint Method evaluation. These datasets were selected because they possessed clearly identifiable cardiac-related artifacts and provided direct comparison between the original Fingerprint Method and our new method. The superior performance of ARCI, particularly the superior sensitivity compared to the Fingerprint Method, was likely due to several factors. In the Fingerprint Method, 14 different spatial, spectral, statistical, and temporal features were evaluated to classify physiological artifacts. Among these 14 features, a CIF, the CIF, was developed to specifically capture the spectral properties of cardiac-related interferences. In the present study, the CIF was retained, and an additional feature was added which exploited the temporal and waveform characteristics of cardiac artifacts, the CorrCI. The present results indicate that the elimination of features which do not disentangle cardiac interference combined with the addition of this new feature, CorrCI, resulted in greater discrimination of cardiac and non-cardiac ICs. Specifically, the combined use of the CIF and CorrCI features led to fewer false cardiac artifact classifications (false positives), which may have contributed to the greater sensitivity, hence reliability, of ARCI.

An additional advantage of including the CorrCI feature is its ability to classify not only electrical cardiac artifactual activity but also pulse-related artifacts. While electrical cardiac and pulse-related interference share common spectral properties (i.e., the same cardiac frequency and IBI), pulse-related artifacts have a unique waveform characterized by a higher amplitude and slower temporal progression than typical electrical cardiac waveforms. Furthermore, pulse-related interference typically affects one or a few EEG channels located near blood vessels. The CorrCI feature exploits the stereotypical waveform characteristics of each hypothetical cardiac IC and is therefore able to capture these unique pulse features. When we specifically examined artifactual ICs of pulse-related origin, we determined that ARCI was able to correctly classify between 10 and 20% more cardiac-related artifactual ICs. This resulted in a greater percentage of true positive classifications compared to the original Fingerprint Method, thereby further contributing to the increased sensitivity of ARCI. To our knowledge, no other studies have reported the effectiveness of their algorithms in removing cardiac-related artifacts of pulse origin. For example, in a recent report by Waser et al. (2017), the authors developed a method based on temporal and spectral variations in EEG time courses to detect several physiological and non-physiological artifacts, including the cardiac one. Their method can detect interference in single EEG channels with an average accuracy of 89.0 ± 2.1, which may be beneficial in the detection of pulse-related activity; however, the authors do not report on pulse artifact identification specifically, and the applicability of their method is reduced by the need for concurrently recorded ECG.

A third factor that may have contributed to the more successful performance of ARCI is the use of the Extended Informax algorithm for the decomposition of EEG signals. The Fingerprint Method used the Infomax algorithm which has been proven effective in isolating noise components related to artifactual activity (Bell and Sejnowski, 1995; Delorme and Makeig, 2004). However, because pulse-related cardiac interference are generally sub-Gaussian, the Extended Infomax algorithm may be more effective at detecting this activity (Vigário et al., 2000). In fact, Extended Infomax has been proven to be better at separating signal sources that have super-Gaussian or sub-Gaussian distributions (Lee et al., 1999; Greco et al., 2008). A variety of ICA algorithms have been utilized to separate cardiac and non-cardiac sources including wavelet-based methods (Castellanos and Makarov, 2006), fastICA (Hamaneh et al., 2014), Infomax (Radüntz et al., 2017; Jafarifarmand and Badamchizadeh, 2018), and Extended Infomax (Mammone and Morabito, 2008; Viola et al., 2009; Frølich et al., 2015). Most of these ICA approaches, combined with other techniques, have been successful in identifying cardiac-related interferences, although it is difficult to assess whether they were more effective than ARCI because the denoised signals were often evaluated by simple visual inspection or by means of correlation-based approaches. Only Frølich et al. (2015) statistically evaluated the effectiveness of their method in detecting cardiac interference. They estimated the balanced accuracy – equal to the mean of specificity and sensitivity in the binary case – which, for groups of testing datasets including more than one dataset, ranged between 0 and 0.85. However, it remains an outstanding question whether successful cardiac artifact identification depends on the number of ICs that are decomposed by a given ICA algorithm. One of our aims in developing ARCI was to ensure that our approach could be applied across a variety of clinical and experimental settings. As such, we sought to determine if ARCI could successfully classify cardiac ICs when data were decomposed into 20 ICs (as typically occurs in clinical applications) or into a greater number of ICs (50 or 80 ICs as occurs in experimental and high-density EEG settings). Results indicate that our method was able to successfully identify cardiac-related interference regardless of the number of ICs and represents an advantage over other approaches where different ICA decompositions were not evaluated.

To provide an additional assessment of the performance of ARCI, we evaluated automatic cardiac artifact classification in eyes-closed EEG datasets where concurrently recorded ECG data were available. When we compared the spectral density properties of the ECG signals with the spectral densities of the automatically classified cardiac ICs, we determined that the classified cardiac ICs were strongly correlated with the true cardiac signal, resulting in an average correlation greater than 0.65. Furthermore, the correlation between ECG and cardiac classified ICs was significantly greater than the correlation with non-cardiac classified ICs, suggesting that ARCI was able to successfully discriminate cardiac and non-cardiac ICs. A number of methods used to classify and remove cardiac interference from EEG signals rely on simultaneously recorded ECG. One common method is ensemble average subtraction (EAS) where an average template waveform is created using the ECG signal and subsequently subtracted from the EEG traces (Nakamura and Shibasaki, 1987; Sahul et al., 1995; Abtahi et al., 2014). Another common method is adaptive filtering, which likewise depends on the ECG signal. In this approach, the ECG trace is used as a reference channel and adaptive filters are applied to cancel the artifact from EEG (Correa et al., 2007; Jafarifarmand and Badamchizadeh, 2013; Navarro et al., 2015). There are several additional variations, including a method developed by Devuyst et al. (2008) that uses an ICA-based method combined with a filtering approach, and a method recently developed by Waser et al. (2018) that identifies electro-cardiogram components using a combination of ICA and autocorrelation. While these methods have generally produced favorable results (e.g., Devuyst and colleagues obtained a correction rate of 91.0%, while Waser and colleagues achieved a detection accuracy of 99%), they are clearly ineffective in situations where concurrent ECG recordings are prohibitive or cumbersome as can occur in sports applications (Thompson et al., 2008). One of the advantages of the ARCI method is that it does not depend on simultaneous ECG recording while achieving comparable or superior results to those obtained when external ECG recordings are considered. One method that does not rely on simultaneous ECG recording was very recently proposed by Dora and Biswal (2019). It is based on the use of a time-frequency approach (the modified S Transform – MST), and successfully detected cardiac electrical interferences (but no pulse contamination) in polysomnographic EEG recordings. However, the statistical analysis provided includes accuracy values that range from 98.61 to 99.05%, which are lower than the accuracy values that we obtained in the detection of cardiac interference with ARCI (ranging from 99.1 to 99.9%).

One issue that should be addressed is the presence of cardiac-related interference in MEG data. Cardiac artifacts contaminate not only EEG, but also present a serious concern in MEG data acquisition and processing (Escudero et al., 2007; Escudero et al., 2011; Breuer et al., 2014a,b; Hasasneh et al., 2018). Indeed, electrical cardiac activity can create greater interference in MEG acquisitions where the insulating properties of the scull do not attenuate the interference (Hämäläinen et al., 1993). On the other hand, contamination due to cardiovascular interference is minimal because MEG sensors are fixed and typically are not in direct contact with the scalp. We hypothesize that ARCI is generalizable to MEG acquisitions, as well. In the present study ARCI was tested using only EEG datasets, and its performance in MEG remains to be demonstrated. In future work, we will validate ARCI for cardiac artifact removal in MEG data.

To classify cardiac-related interference, we employed features that modeled the hypothetical cardiac frequency across a broad spectrum of typical resting cardiac frequencies (Opthof, 2000; Azevedo et al., 2014). While it is unlikely that this frequency range would capture higher cardiac frequencies encountered during sports activity, the frequency range can be easily adjusted to apply the ARCI method to a wide range of acquisition tasks. Another issue that may arise is the wide variability in the cardiac frequencies encountered during acquisitions including periods of activity and periods of rest. In such situations, it is likely that more advanced adjustments would have to be made to account for this wide variability. This can include time segmented ICA methods that do not violate stationarity assumptions (Onton and Makeig, 2006), or the epoching of activity and rest intervals to differentiate suitable frequency ranges that could capture cardiac interference at different heart rates. We are currently developing the ARCI method to permit adjustments of hypothetical cardiac frequencies and applying the ARCI method to EEG data acquired during sports performance tasks. We will report these results in a subsequent publication.

Our new method for the automatic classification and removal of cardiac-related interference from EEG data offers multiple advantages over existing methods: applicability in both clinical and experimental settings, including sports science applications, thanks to insensitivity to IC decomposition level; valid artifact classification without the need for simultaneous ECG data collection; the ability to successfully classify interferences generated not only by the electrical activity of the heart but also due to cardiovascular dynamics. Overall, ARCI represents a significant step in the automatic detection and removal of cardiac-related EEG interferences.

## Ethics Statement

This study was carried out in accordance with the recommendations of the Guidelines for Sperimentazione clinica non-farmacologica no profit, monocentrica, University G. d’Annunzio of Chieti-Pescara (Italy) with written informed consent from all subjects. All subjects gave written informed consent in accordance with the Declaration of Helsinki. The protocol was approved by the University G. d’Annunzio of Chieti-Pescara (Italy) with Ethical Application Ref. n.10-21/05/2015.

## Author Contributions

GT designed the procedure for the new method, developed the software, analyzed the data, and collected the eyes-closed EEG datasets. GT and DS wrote the manuscript. SC supervised all phases of the work. All co-authors evaluated the results, revised the manuscript, and contributed to the study design.

## Conflict of Interest Statement

The authors declare that the research was conducted in the absence of any commercial or financial relationships that could be construed as a potential conflict of interest.

## Supplementary Material

Figure S1 Layout of the caps used for the acquisition of the eyes-closed EEG datasets: **(A)** the dry electrode cap with 64 multipin polyurethane electrodes with a Ag/AgCl coating, arranged in a quasi-equidistant montage; **(B)** the commercial wet cap with 64 Ag/AgCl electrodes in an adapted 10-10 montage (Waveguard, Advanced Neuro Technologies B.V., Enschede, Netherlands).
