# Supplementary figures and images for: Automatic Removal of Cardiac Interference (ARCI): A New Approach for EEG Data
Source: Front Neurosci. 2019 May 8;13:441. doi: 10.3389/fnins.2019.00441 (PMC6517508; doi:10.3389/fnins.2019.00441)

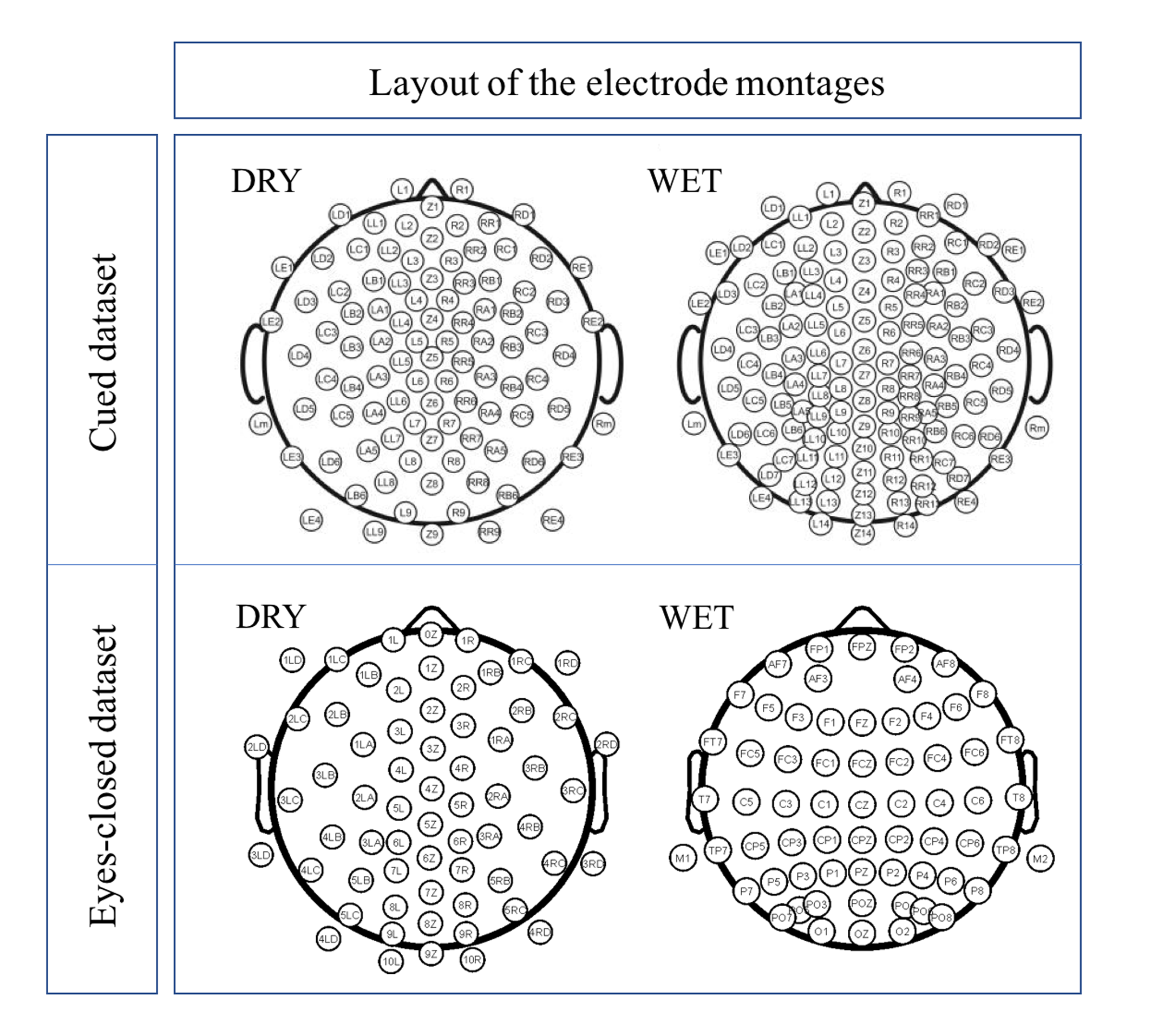

Supplement: Figure S1 — Layout of the caps used for the acquisition of the eyes-closed EEG datasets: (A) the dry electrode cap with 64 multipin polyurethane electrodes with a Ag/AgCl coating, arranged in a quasi-equidistant montage; (B) the commercial wet cap with 64 Ag/AgCl electrodes in an adapted 10-10 montage (Waveguard, Advanced Neuro Technologies B.V., Enschede, Netherlands). [file Image_1.tif]
